# Supplementary material for: Causes and consequences of sex-chromosome turnovers in Diptera
Source: Evol Lett. 2026 Mar 12;10(3):253–68. doi: 10.1093/evlett/qrag003 (PMC13231274; doi:10.1093/evlett/qrag003)
Supplement: qrag003_Supplemental_Files [file qrag003_supplemental_files.zip › Supplementary_Datasets.zip!/Supplementary_Datasets/Dataset_2_Graphical_representation_outgroups/Culicidae_Anopheles_atroparvus_chromosomes.pdf]

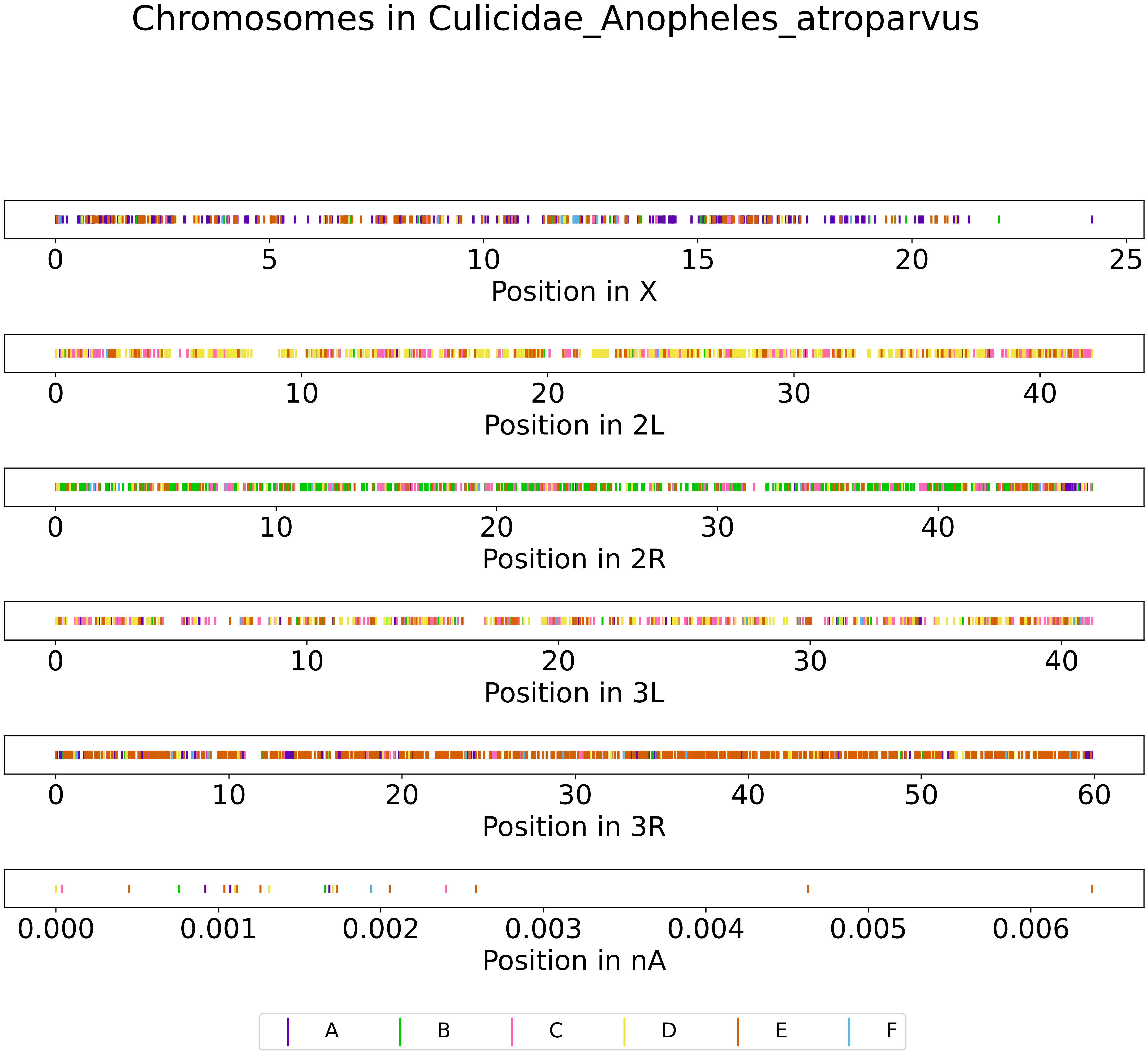
Chromosomes in Culicidae_Anopheles_atroparvus
0 5 10 15 20 25
Position in X
0 10 20 30 40
Position in 2L
0 10 20 30 40
Position in 2R
0 10 20 30 40
Position in 3L
0 10 20 30 40 50 60
Position in 3R
0.000 0.001 0.002 0.003 0.004 0.005 0.006
Position in nA
A
B
C
D
E
F
